# Phase Behaviour, Functionality, and Physicochemical Characteristics of Glycolipid Surfactants of Microbial Origin

**DOI:** 10.3389/fbioe.2022.816613

**Published:** 2022-01-27

**Authors:** Karina Sałek, Stephen R. Euston, Tomasz Janek

**Affiliations:** ^1^ Institute for Life and Earth Sciences, School of Energy, Geoscience, Infrastructure and Society, Heriot-Watt University, Edinburgh, United Kingdom; ^2^ Institute of Biological Chemistry, Biophysics and Bioengineering, School of Engineering and Physical Sciences, Heriot-Watt University, Edinburgh, United Kingdom; ^3^ Department of Biotechnology and Food Microbiology, Wrocław University of Environmental and Life Sciences, Wrocław, Poland

**Keywords:** biosurfactants, glycolipids, rhamnolipids, sophorolipids, lipopeptides, global use of surfactants

## Abstract

Growing demand for biosurfactants as environmentally friendly counterparts of chemically derived surfactants enhances the extensive search for surface-active compounds of biological (microbial) origin. The understanding of the physicochemical properties of biosurfactants such as surface tension reduction, dispersion, emulsifying, foaming or micelle formation is essential for the successful application of biosurfactants in many branches of industry. Glycolipids, which belong to the class of low molecular weight surfactants are currently gaining a lot of interest for industrial applications. For this reason, we focus mainly on this class of biosurfactants with particular emphasis on rhamnolipids and sophorolipids, the most studied of the glycolipids.

## 1 Introduction

There is a high demand for surfactants across almost every sector of modern industry as they are found in a wide range of household products. The global consumption of surfactants increases every year and its global production is expected to reach US$ 28.8 billion until 2023 (Kaczerewska et al., 2020). Surfactants are used extensively in the household and personal care market, with the latter constituting approximately 14% of the global surfactant use, with the Asia-Pacific region being the largest consumer (Costello 2018). Other uses of surfactants cover the oil and gas industry, transport, industrial clean-up processes and the food industry (Shukla 2016; Costello 2018).

Many surfactants used in industry are produced synthetically from petrochemical feedstock, which is neither sustainable nor environmentally friendly. Additionally, synthetic surfactants are recognized as being relatively eco-toxic and of low biodegradability. This has led to an interest in biosurfactants as green solutions that are environmentally friendly by virtue of their low toxicity and biodegradability compared with their synthetically derived counterparts. According to Global Market Insights, Inc. (Global Market Insights Inc. 2018), by 2024 the biosurfactant demand in personal care market should reach USD 300 million, while the general U.S. biosurfactant market size may exceed even USD 600 million and for the United Kingdom the estimates are as high as USD 210 million. Due to the high demand for cleaning products (both domestic and industrial) detergent use of biosurfactants is expected to dominate the global biosurfactant applications by 2022 (Research and Markets 2017).

Despite the ever-increasing demand, the commercial production of biosurfactants is still a challenge due to high raw material costs. About 30–50% of the total cost comes solely from the preparation of culture medium for biosurfactant production secreted by microorganisms (Ebadipour et al., 2016). Thus, current research challenges include a decrease in the cost of raw materials, consumables, utilities, labor, and waste treatment and disposal (Bjerk et al., 2021). In terms of price, synthetic surfactants (priced at approximately USD 2/kg) (Kosaric and Vardar-Sukan 2015), are much cheaper compared to the pure rhamnolipids (priced range between USD 1250/kg and USD 2500/kg) (www.agaetech.com and www.sigmaaldrich.com (accessed date: 27 December 2021)). Glycolipid biosurfactants such as lactonic sophorolipids cost around USD 55 000/kg (www.carbosynth.com (accessed date: 27 December 2021)), whereas the cost of lipopeptide biosurfactant surfactin is USD 17 600/g (www.sigmaaldrich.com (accessed date: 27 December 2021)), since it is produced in lower quantities. Compared to pure commercially available biosurfactants, Ebadipour et al. (2016) described production of rhamnolipids (24 g/L) with corn steep liquor at an estimated price of USD 12/kg. According to the work of Ashby et al. (2013) production of sophorolipids using glucose and high oleic sunflower oil cost of USD 2.54/kg. According to Singh et al. (2019) cost reduction using cheaper raw materials, or agroindustrial wastes, could lead to a cost being closer to the lucrative 2 USD/kg for commercial surfactants.

Owing to the increasing customer awareness and demand for natural, vegan, organic and sustainable food additives, biosurfactants seem to represent a good candidate to replace their synthetic counterparts in food industry (Nitschke and Silva 2018). A well-studied antimicrobial and antibiofilm potential of biosurfactants enables them to be used as antiadhesive and biofilm-disrupting agents as well as food preservatives (Sambanthamoorthy et al., 2014; Nitschke and Silva 2018; Sałek and Euston 2019). Another opportunity for biosurfactant use in food industry lies within their surface-active properties, making them potentially highly valuable emulsifiers and emulsion stabilisers in not only food, but also cosmetic and pharmaceutical industry, where emulsions (especially nanoemulsions) are used as drug-delivery systems (Dalgleish 2006; Bai and McClements 2016).

Glycolipids biosurfactants have the most potential in industry with rhamnolipids and sophorolipids generating the most interest especially in detergency. Therefore, this review article focuses on the glycolipid-based biosurfactants, their functional properties, structure and applications.

## 2 Classification of Biosurfactants of Microbial Origin

Biosurfactants are classified according to molecular weight–low or high. Glycolipids, flavolipids and lipopeptides are low molecular weight (LMW) biosurfactants while lipoproteins, polysaccharides and lipopolysaccharides belong to the high molecular weight (HMW) class (Smyth et al., 2010). Currently, the LMW biosurfactants, especially glycolipids are of high interest for exploitation in the detergent and personal care markets, but have potential in the cosmetic, biomedical and food industries. A review of the major LMW biosurfactants follows in the subsequent sections, with a particular emphasis on glycolipids.

### 2.1 Glycolipids

Glycolipids are biosurfactants with a range of structures, made up of a sugar polar group and a lipid group. This diverse group of surfactants includes rhamnolipids, sophorolipids (including their derivatives), mannosylerythritol lipids, trehalolipids and cellobioselipids (Mulligan 2005; Franzetti et al., 2010).

A slightly different group of microbial lipids–polyol lipids, produced by yeast and fungi, are also classified as glycolipids. The two main categories of polyols–liamocins and polyol esters of fatty acids (PEFA) are less studied than the other glycolipids, however, they may present a good potential for commercialisation (Garay et al., 2018).

### 2.2 Lipopeptides

Lipopeptides (LPs) are a group class of biosurfactants containing a variable length fatty acid and cyclic peptide polar group. Several isoforms have been identified, with surfactin, iturin, fengycin, lichenysin among the most common, with some microorganisms excreting more than one isoform (Mnif and Dhouha 2015b). Bacteria, yeasts, molds and actinomycetes have been identified as lipopeptides producers (Mnif and Dhouha 2015b). Surfactin, a secondary metabolite of the bacterium *Bacillus subtillis*, is the most studied (Mnif and Dhouha 2015b), with reported anti-microbial and anti-mycoplasma activities, as well as cell lysate promoter and fibrin clotting properties (Chen et al., 2015). *Bacillus* sp. also produce lichenysin, iturin and fengycin, while *Pseudomonas* sp. secrete tensin, pseudofactin and viscosin. Important LP antibiotics are produced by *Streptomyces* sp. (amphotericin and laspartomycin) and *Pseudomonas* sp. (polymyxin). Amphotericin and lasapartomycin can be used as therapies for difficult to treat aspergillosis and candidiasis fungal infections as well as *Leishmania* parasites (Caffrey et al., 2001; Tollemar et al., 2001). Laspartomycin can treat antibiotic-resistant enterococci and *Staphylococcus aureus* (Borders et al., 2007; Strieker and Marahiel 2009).

Lipopeptide function is linked to their surface chemistry, with their antibiotic activity being due to their ability to adsorb to and insert into cell membranes (Carrillo et al., 2003). Lichenysin is reported to be the most surface active of the lipopeptides, with a CMC half that of surfactin (Yakimov et al., 1995), a characteristic attributed to a less polar peptide head group and longer lipid tail. Lichenysin is also reported to be highly heat, pH and salt stable (McInerney et al., 1990). The self-assembly of lipopetides such as surfactin and iturin has been studied as this will influence surface behaviour and biological activity. Surfactin has been reported to have a low critical micelle concentration (CMC) in the range 7.5–10 μM (Heerklotz and Seelig 2001; Carrillo et al., 2003), and iturin in the range 25–40 μM (Harnois et al., 1988; Maget-Dana and Peypoux 1994). Surfactin also forms unusually small micelles (Shen et al., 2009). More complex self-assembled structures have been observed, for example for mycosubtilin produced by *Bacillus spp*. In this lipopeptide extended nanotape structures (Hamley et al., 2013) form rather than micelles. There is a recognition that lipopeptide phase behaviour has not been studied in the same detail as other biosurfactants (Hamley 2015).

### 2.3 Flavolipids

Flavolipids, first described by Bodour et al. (2004), are a relatively newly identified class of biosurfactants from the genus *Flavobacterium*. The surfactant isolated by the authors had a critical micelle concentration (CMC) of 300 mg/L and reduced surface tension to 26 mN/m. A strong emulsifying ability of the surfactant and successful mineralization of hexadecane was also reported. The hydrophilic moiety of flavolipids is a citric acid and two cadaverine molecules, which is unlike other lipid-based surfactants.

The ability of flavolipids to form micelles and also vesicles in the presence of iron (Martinez et al., 2000; Serrano Figueroa et al., 2016) hints at complex phase behaviour, but as far as we can determine this has not been studied in any detail.

## 3 Glycolipids

The most studied group of LMW biosurfactants are the glycolipids and there is growing interest in their application for industrial applications due to this. There follows a detailed description of selected glycolipids and recent advances in their applications. Table 1 presents a brief summary of recent reports on the activity and potential application of glycolipids.

**TABLE 1:** Recent reports on possible applications glycolipids.

Biosurfactant	Biological or Physicochemical activity	Industrial sector	References
Mannosyl erythritol lipids (MELs)	Anti-melanogenic	Cosmetic	Bae et al. (2018)
MELs	Anti-bacterial	Foods	Shu et al. (2019)
		Medical	
MELs	Anti-bacterial	Medical	Ceresa et al. (2020b)
		Food	
MELs	Nanoparticles	Pharmaceutical	Bakur et al. (2019)
Sophorolipids	Biofilm inhibition and disruption	Pharmaceutical	Díaz De Rienzo et al. (2016)
		Food	
		Packaging	
Acidic sophorolipids	Anti-bacterial activity	Medical	Lydon et al. (2017)
Free acid and lactonic sophorolipids	Antimicrobial activity and inactivation mechanism against pathogenic *Escherichia coli* O157:H7	Medical	Zhang et al. (2017)
		Pharmaceutical	
		Food	
Lactonic sophorolipids	Solid-lipid nanoparticles	Pharmaceutical	Kanwar et al. (2018)
Sophorolipids	Anti-HIV activity	Medical	Shah et al. (2005)
	Spermicidal activity		
Sophorolipids	Anti-bacterial	Medical	Díaz De Rienzo et al. (2015); Díaz De Rienzo et al. (2016); Ceresa et al. (2020a)
	Anti-biofilm	Food	
		Biomaterial	
Sophorolipids	Anti-cancer (breast adenocarcinoma lines MDA-MB-231)	Medical	Ribeiro et al. (2015)
Sophorolipids	Anti-cancer	Medical	Shao et al. (2012)
Sophorolipids	Nanoparticle synthesis	Medical	Singh et al. (2009); Shikha et al. (2020)
Mono & di-RLs	Anti-microbial activity	Medical	Ndlovu et al. (2017)
		Food	
Mono & di-RLs	Cytotoxic effect on human breast cancer cells	Medical	Rahimi et al. (2019)
Rhamnolipids	Stabilisation of oil in high water internal phase emulsions (HIPEs)	Food	Dai et al. (2019)
		Medical	
		Personal care Cosmetics	
Rhamnolipids	Anti-bacterial activity towards food pathogens: *B. cereus* and *L. monocytogenes*, *S. aureus*	Food	de Freitas Ferreira et al. (2018)
		Packaging	
Rhamnolipids	Nanoemulsions for drug delivery mechanism against SCC7 tumour cells	Medical	Yi et al. (2019)
Rhamnolipids	Biodegradation of hydrophobic organic compounds	Bioremediation strategies	Zeng et al. (2018)
Rhamnolipids	Microbial-enhanced oil recovery (MEOR)	Environmental protection	Câmara et al. (2019)
		Petroleum	
Rhamnolipid	Nanoparticle synthesis	Medical	Kumar et al. (2010); Bharali et al. (2013); Saikia et al. (2013); Kumar and Das (2018); Bayee et al. (2020)
Trehalolipids	Anti-microbial	Medical	Janek et al. (2018)
	Anti-adhesive	Biomaterials	
Trehalolipids	Immunomodulato-ry and membrano- tropic activity	Medical	Kuyukina et al. (2015); Kuyukina et al. (2020)
Trehalolipids	Anti-tumour	Medical	Christova et al. (2015)

### 3.1 Rhamnolipids

Rhamnolipids (RLs) originally identified, as sugar lipids produced by the bacterium *Pseudomonas aeruginosa,* an opportunistic pathogen bacterium are one of the best-known biosurfactants. Rhamnolipids were first mentioned as novel biomolecules in 1946, when the antibacterial activity of “pyolipic acid,” from *P. pyocyanea* (later *P. aeruginosa*) against *Mycobacterium tuberculosis* (Bergström et al., 1946) was reported. Three years later Jarvis and Johnson isolated and identified pyolipic acid as “an acidic, crystalline glyco-lipide” (Jarvis and Johnson 1949).

#### 3.1.1 Structure and Production

The structures of RLs can vary significantly depending on the producing microorganism, carbon source and culture conditions. In general, RLs can be described as mono- (one rhamnose ring) or di-rhamnolipids (two rhamnose rings connected via an *α*-1,2-glycosidic bond). The lipid part has one, two or three saturated or unsaturated *ß*-hydroxy fatty acids with varied chain length (C_8_-C_16_) (Lang and Wullbrandt 1999; Gunther IV et al., 2005; Abdel-Mawgoud et al., 2010).

The best-described microorganism producing rhamnolipids is *Pseudomonas aeruginosa*. Production of RLs by this organism has undergone extensive process and genetic optimization and scale-up to the point where RL products are commercially available. Despite very good functional features of RLs, the fact that they are produced by a pathogenic strain limits their applications in many consumer industries e. g personal care, food and cosmetics. Therefore, an extensive search for other, non-pathogenic RL-producers continues. Several non-pathogenic *Pseudomonas* species produce rhamnolipids, such as *P. alcaligenes* (Oliveira et al., 2009), *P. fluorescens* (Abouseoud et al., 2008), *P. chlororaphis* (Gunther IV et al., 2005), *P. putida* (Wittgens et al., 2011), and *P. stutzeri* (Celik et al., 2008; Singh and Tripathi 2013). Apart from *Pseudomonas* species, other species of bacteria such as *Burkholderia plantarii* (Andrä et al., 2006), *Burkholderia thailandensis* (Elshikh et al., 2017), *Acinetobacter calcoaceticus* (HoŠková et al., 2013; Hošková et al., 2015), *Enterobacter asburiae* (HoŠková et al., 2013; Hošková et al., 2015), and *Marinobacter* sp. (Voulgaridou et al., 2021) are also known to produce RLs.

The fermentation conditions as well as the strain selection are important for the production of the particular RLs and the number of homologues in the product. These can vary significantly even within the same species. Abalos et al. (2001) identified seven homologues of RLs isolated from *P. aeruginosa* AT10 grown on soybean oil refinery wastes. Two compounds–Rha-C_10_-C_10_ and Rha-Rha-C_10_-C_10_ were predominant in the isolated mixture and were characterised with very high anti-fungal activity. A total number of 15 rhamnolipid homologues in a post-fermentation mixture of *P. aeruginosa* 47T2 NCBIM 40044 (grown on waste cooking oils) were identified by Haba et al. (2003). Also, in this example, the most dominant rhamnolipid was Rha-Rha-C_10_-C_10_ (34% relative abundance), followed by Rha-C_10_-C_10_ (19% relative abundance) and Rha-Rha-C_10_-C_12_ (20% relative abundance). Gong et al. (2015) presented a comparison of the yields of isolated rhamnolipids when *P. aeruginosa* TIB-R02 was grown on eight different food oil carbon sources. The di-RL Rha-Rha-C_10_-C_10_ was identified as the most abundant (>40% relative abundance) homologue in the case of six tested oils (coconut, peanut, olive, palm, grapeseed and soybean oil), while Rha-C_10_-C_10_ was the most dominant (>40%) for corn and frying oils. Rha-Rha-C_12_-C_10_ was the third most abundant homologue present in all of the isolated mixtures, but its content did not exceed 20% in any of the cases (Gong et al., 2015).

Some researchers have developed methods for the chemical synthesis of RLs to overcome perceived issues with scaled up biological production (Coss et al., 2012; Palos Pacheco et al., 2017, Palos Pacheco et al., 2021; Compton et al., 2020). Whilst these can produce RLs at commercial scale and are marketed as green surfactants (www.glycosurf.com), the methods are not yet seen as economically viable compared to other synthetic surfactants (Palos Pacheco et al., 2021). They are also bio-inspired (synthetic) and not truly biological methods for RL production and thus are likely to suffer from the consumer perception that they are synthetic and not natural, even if as claimed the methods are green (Palos Pacheco et al., 2021). Scientifically, these synthesis methods have proven useful for the production of specific RL congeners that allow the effect of congener structure on surface active properties to be studied (Palos Pacheco et al., 2021).

#### 3.1.2 Phase Behaviour

Surfactants have long been known to display complex phase behaviour in aqueous solution owing to their ability to self-assemble into various structures such as micelles, vesicles, bilayers and various liquid crystalline mesophases depending on concentration and temperature (Tadros 2005). The phase behaviour impacts on properties of the surfactants in industrial applications. Rhamnolipids, and glycolipids in general, also display this rich phase behaviour. Glycolipids, including RLs, at concentrations around the critical micelle concentration (CMC) form micelles that are spherical, disk-like or rod-like (Söderman and Johansson 1999).

Many studies characterise only the CMC of the rhamnolipids, although the micelle is only one of the potential self-association structures formed. Nitschke et al. (2011) compared CMC and surface tension (ST) of rhamnolipids isolated from various *Pseudomonas* species. According to this study, the lowest ST was for RLs isolated from *P. chlororaphis*, and varied between 25 and 30 mN/m. The RLs from *P. aeruginosa* were reported to lower ST to 27.3 mN/m and have a CMC of 13.9 mg/L. Close to that range were RLs isolated from *P. alcaligenes* with ST equal to 28 mN/m and CMC 30 mg/L. The highest ST among *Pseudomonas* species was detected for *P. fluorescens*–35 mN/m and CMC reaching 20 mg/L. In general, the CMC of rhamnolipids can vary even between 10 and 200 mg/L depending on the producing microorganisms, carbon sources, medium composition and other factors such as temperature, aeration and pH (Nitschke et al., 2005). In practice, it has been known for some time that self-association in rhamnolipids is more complicated than just formation of micelles. Both Ishigami et al. (1987) and Champion et al. (1995) observed using cryo-TEM that rhamnolipids formed either lamellar, micelle or vesicle phases depending on the pH. Similarly, Sánchez et al. (2007), report a concentration-dependent micelle-to-vesicle transformation for diRLs at pH 7.4. Small angle neutron scattering (SANS) has revealed that both the monorhamnolipid R1 (Rha-C_10_-C_10_) and dirhamnolipid R2 (Rha-Rha-C_10_-C_10_) form small, globular, elliptical core shell micelles with the ratios of the minor-major axes in the range 1:2–10 (Chen et al., 2010) and average aggregation numbers (number of rhamnolipids per micelle) of *n* = 50 for R1, *n* = 30 for R2 and *n* = 40 for a mixed R1/R2 micelle. The reduced packing efficiency of the two rhamnopyranose rings in the headgroup of R2 leads to a lower curvature of the micelle surface, and so R2 micelles are more asymmetric than those of R1. Palos Pacheco and co-workers (Palos Pacheco et al., 2021) have been able to carry out detailed studies of the micellization and aggregate structure of a homologous series of monorhamnolipids with a single C14 chain, and second chain lengths of 6, 8, 10, 12 and 14 carbons. This has allowed them to study the intricacies of RL congener structure on surface properties. Surprisingly, they find that the CMC does not vary linearly with chain length. The CMC is lowest for RlC_14_C_10_ and increases for smaller and higher second lipid chain lengths. The minimum surface tension mirrors this behaviour. This behaviour suggests that interactions within the RL congener and between molecules at the interface is complex and plays a significant role in their surface behaviour. Molecular dynamics simulations of RL congeners with differing chain length (C_14_C_14_, C_14_C_10_ and C_10_C_14_) also show differing properties of the congeners suggesting complex surface interactions within the molecule (Euston et al., 2021).

Molecular simulation has been used to investigate the internal structure of RL micelles and biosurfactants in general (Euston 2017) in more detail. Schwartz and co-workers have carried out a detailed study of the structure of micelles and vesicles for both mono and di-rhamnolipids (Munusamy et al., 2017). They found that for nonionic R1, spherical, ellipsoidal, torus-like, and unilamellar vesicle structure can form depending on aggregation number (Eismin et al., 2017), with anionic R1 also forming long tubular structures (Luft et al., 2020). For non-ionic R1, the aggregation number for the micelles did not go above about *n* = 40, with this limited by a lack of H-bonding and a restricted size for the hydrophobic core due to the relatively small C10 alkyl chains. When a charge is present on the anionic R1, larger micelles can form due to H-bonding altering the packing of the molecules. Simulations of R2 di-rhamnolipids support the experimental evidence that they form smaller micelles (around *n* = 22) because of the presence of a second rhamnose group, although increasing the length of the akyl chain allows for larger micelle aggregation numbers. For di-rhamnolipids, simulation (dissipative particle dynamics, DPD) supports the formation of more complex phases at high biosurfactant concentration (Xu et al., 2018). Three multilayer structures of micelles were observed at pH < 4 depending on the concentration. At concentrations below 10% wt of diRLs, bi-layer spherical micelles formed, with their diameter increasing with increased concentration. Further increase in concentration to 15–20 wt% resulted in the formation of bi-layered rod-like micelles, which changed into net-like bi-layered lamellar phases at 25% wt. A double bi-layer was obtained when the concentration reached 40 wt% (Xu et al., 2018). At pH above 7.4, novel anisotropic morphologies were discovered. Starting from 5 to 10%, patchy spherical micelles were observed. At 11%, rod-like micelles with a helical pattern formed, and at 15% a net-like lamellar phase. Finally, the formation of vesicles was observed at concentration reaching 40 wt% (Xu et al., 2018).

This research shows that RLs have the potential to be used in preparation of advanced functional systems such as anisotropic nanomaterials, which can be applied in biomaterials, drug delivery systems, microelectronics, optoelectronics, sensors or tissue engineering (Xu et al., 2018, Xu et al., 2020).

#### 3.1.3 Physicochemical Characterisation and Functionality

If rhamnolipids are to replace conventional surfactants in industry, their physicochemical and functional properties need to be comparable to those they replace. Pekdemir et al. (2005) studied the emulsifying ability of rhamnolipids compared to other biological surfactants (tannins, saponins, lecithin). Rhamnolipids, like all other surfactants tested, produced oil-in-water emulsions that were unstable and separated within 2 min. Rhamnolipid showed the best emulsifying properties in both distilled water and seawater. Lovaglio et al. (2011) have looked at the pH dependence of rhamnolipid emulsification. They confirm the emulsifying ability of rhamnolipids and show that the optimum properties are at basic pH, and that under these conditions rhamnolipids have as good an emulsifying ability and stability as the common anionic surfactant sodium dodecyl sulphate. Bai and McClements (2016) have noted that rhamnolipids can be used to form very fine (<0.15 µm) oil droplets with medium chain triglyceride oils and also with longer chain corn and fish oils at low surfactant to oil ratios (<1:10). Furthermore, the emulsions were stable over a wide range of pH (5–9), salt content (up to 0.1 M NaCl) and temperature (up to 90°C). Instability was observed under highly acidic conditions (pH 2–4) and high ionic strength (NaCl 0.2–0.5 M). This was explained in terms of the decrease in electrostatic repulsion between droplets at low pH (loss of charge) and at high salt (charge screening effects). Özdemir et al. (2004) have also observed that a reduced charge repulsion at low pH leads to more compact interfacial adsorbed layers of rhamnolipids, and alters the CMC, which will also change the adsorption properties. A similar effect is seen with salts (Helvaci et al., 2004) where increasing NaCl concentration up to 0.5 M leads to more dense packing of rhamnolipids at the air-water interface. Rhamnolipids are also able to emulsify vegetable oils more efficiently than hydrocarbons (Janek et al., 2013), and can form emulsions with essential oils, thus offering a way to deliver the bioactive properties of plant extracts (Haba et al., 2014). Russell et al. (2021), have looked at the combined emulsifying ability of rhamnolipids with egg white protein (EWP). They noted that increasing the RL concentration in an emulsion homogenised at constant EWP content led to a large decrease in emulsion droplet size. In contrast, when lecithin or monoglyceride was substituted for the RL, these surfactants had little effect on droplet size. This suggests that RL interacts with protein at the interface in a different way to lecithin and monoglyceride.

Rhamnolipid adsorption at the air-water interface and the relationship to foaming properties has also been studied. Khoshdast et al. (2012) compared the ability of rhamnolipid to other common foaming agents to form froth in froth flotation separation of minerals in mining operations. The rhamnolipid outperformed all tested frothers, which was explained by its greater ability to lower surface tension and the higher interfacial elasticity due to strong inter-rhamnolipid interactions in the adsorbed film. The high surface elasticity of the rhamnolipid, however, limits foaming ability under certain conditions. During foaming through air sparging rhamnolipids only form foams between certain limiting air flow rates (Özdemir et al., 2004). Below the lower flow rate limit, foam cannot form because the high surface elasticity of the monolayer rhamnolipid resists surface film expansion. Above the upper limit of airflow, the rigid surface film fractures because the applied stress is too high, and the foam collapses immediately. The same authors also looked at the foaming properties of the individual mono- and di-rhamnolipids R1 and R2. R1 is a better foaming agent than R2 and both show a pH dependence in foaming properties, with R1 more sensitive to pH. Özdemir et al. (2004) explain this as being due to a greater screening of the carboxyl group by the two sugar groups in R2.

Despite foam being a desired feature and determinant of the commercial value of many products (especially personal care ones), making RLs a perfect candidate for commercialization, it also can be a limiting factor in the process (Rieger 1996; Bator et al., 2020). Extensive foaming poses a real challenge in sustaining high product yields along with the low production costs (Sodagari et al., 2018). Chen et al. (2010) have carried out a detailed neutron reflectivity study of the adsorbed R1 and R2 conformations. Both R1 and R2 behave as classical surfactants with a Langmuir-type isotherm. The surface area occupied per molecule is 60 Å^2^ for R1 and 75 Å^2^ for R2, with the latter reflecting the greater size of the di-rhamnolipid head group. When mixtures of R1 and R2 adsorb to the air-water interface, the R2 molecules are not able to compete efficiently due to steric hindrance from the larger head group, and so a mixed interface is dominated by adsorbed R1.

Due to their physicochemical and functional characteristics, rhamnolipids have already been used in many branches of industry. A lot of research has been devoted to rhamnolipid influence on the biodegradation of petroleum hydrocarbons (Nitschke et al., 2011; Kaczorek et al., 2013). This surfactant-enhanced process was extensively studied in the 90’s, due to the discovery of the ability of *Pseudomonas aeruginosa* to assimilate both aromatic (including PAH–polycyclic aromatic hydrocarbons) and aliphatic hydrocarbons. Zhang and Miller reported the rhamnolipid enhanced dispersion and biodegradation of octadecane (Zhang and Miller 1992, Zhang and Miller 1994), while Deziel et al. (1996) and Arino et al. (1998) reported the growth of *P. aeruginosa* on PAH showing also the involvement of rhamnolipids in the biodegradation of those compounds. Interestingly, the use of rhamnolipids in bioremediation strategies is still investigated and due to the large amounts of reports in this topic, only a few crucial papers are mentioned.

The latest research by Fernández-Peña et al. (2020) presented the impact of four rhamnolipids (mono-RL (C10), mono-RL (C14), di-RL (C10) and di-RL (C14)) on the adsorption of hair-conditioning polymers on damaged hair in order to verify whether glycolipids such as RLs could be a good replacement for chemically derived, commercial surfactants such as SLES. The study showed that RLs increased a degree of surface coverage of surfactant-polymer composition thus enhancing the deposition and hydration efficacy on damaged hair, especially with regards to mono-RL (C10) (Fernández-Peña et al., 2020). The positive outcomes of this research suggest that rhamnolipids of short alkyl chains could be used in hair cosmetics as a replacement for SLES.

Other commercial application of rhamnolipids is in pharmaceuticals, personal care, household cleaning, petroleum recovery, pesticides and biofungicides (Sajna et al., 2015). One area that has received attention is in the synthesis of therapeutic nanoparticles, and in particular gold and silver nanoparticles (Kumar et al., 2010; Bharali et al., 2013; Saikia et al., 2013; Kumar and Das 2018; Bayee et al., 2020). Gold nanoparticles find application in the biomedical field as delivery systems for therapeutic agents, and as sensor systems in diagnostic (typically lateral flow) tests (Nejati et al., 2021). Chemical synthesis of nanoparticles requires reduction of gold (Au^3+^ or Au^+^) or silver (Ag^+^) to the ground state metal by reduction (Iravani et al., 2014; Daruich De Souza et al., 2019). A common reaction is the reduction of HAuCl_4_ using the powerful reducing agent sodium borohydride. To stabilize the nanoparticles and to prevent aggregation, capping agents are added to the reaction mixture which are often surfactants. Synthetic surfactants such as cetyltrimethylammonium bromide adsorb to the nanoparticle surface and stabilize them though charge repulsion. Finding new, green synthesis methods has become a priority as many of the reducing agents and surfactants used are toxic, which presents problems when used for biomedical applications. Green synthesis methods where a single molecule can act as both reducing and capping agents are desirable. Rhamnolipids have been shown to be an effective stabilizing agent for nanoparticles but still require a reducing agent, thus only giving a partial green solution.

### 3.2 Sophorolipids

**FIGURE 1:**
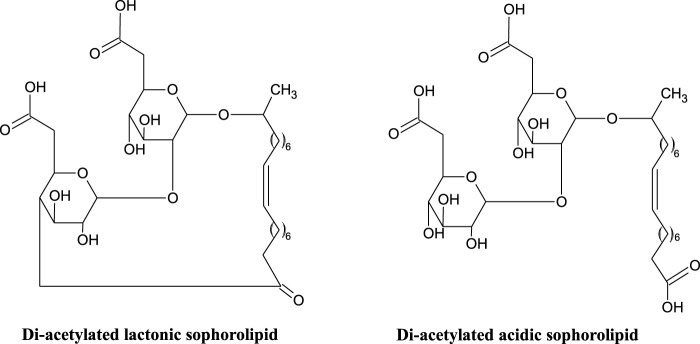
Structure of di-acetylated lactonic and acidic sophorolipids (Hardin et al., 2007; Jezierska et al., 2018).

Sophorolipids (SLs) (Figure 1), first identified in the 1960’s (Gorin et al., 1961), are a glycolipid with a sophorose polar group (two glucose rings linked *via* a β1-2 glycosidic bond) and a hydroxylated fatty acid lipid tail (C18 for those produced by *S. bombicola*). A number of modified sophorolipids including branched sophorolipids (Tulloch et al., 1968; Baccile et al., 2017b); acetylated sophorolipids (Baccile et al., 2017a); bolaform sophorolipids and sophorosides (Van Renterghem et al., 2018); petroselinic sophorolipids (Delbeke et al., 2016a) and chemically modified sophorolipids (Cuvier et al., 2014; Baccile et al., 2016, Baccile et al., 2019; Delbeke et al., 2016b, Delbeke et al., 2018, Delbeke et al., 2019) have been produced and studied, and will also be discussed here.

#### 3.2.1 Structure and Production

Lactonic and acidic (Figure 1) are the two general forms of sophorolipid, secreted by non-pathogenic yeasts such as *Candida, Starmerella* and *Pseudohyphozyma* (Jezierska et al., 2018). These general structures are further differentiated by differing degrees of acetylation. The non-pathogenic nature of the producing organisms is advantageous for food and cosmetic application (Jezierska et al., 2018). Like the rhamnolipids, mixtures of sophorolipid congeners are produced (up to 20), but only a small number are found in significant quantities (Claus and Van Bogaert 2017).

The optimised conditions for production of sophorolipids depend on the selected strain, with some factors common to all. The suggested growth temperature of *Starmerella (Candida) bombicola* ATCC 2214 (the most commonly used strain to produce sophorolipids) is 28.8°C and pH of 3.5 in the bioreactor (Van Bogaert et al., 2007). Since yeasts are very susceptible to any oxygen limitations, especially during the exponential growth phase, a constant oxygen supply must be provided throughout the entire fermentation process (Van Bogaert et al., 2007). Interestingly, the carbon source used in the fermentation is usually a mixture of hydrophilic and hydrophobic compounds, where glucose or glycerol are commonly used as hydrophilic matter. The selection of a hydrophobic carbon source, however, is a more complicated decision, since it can enhance the production of particular type of sophorolipids over the other types. For example, the use of long-chain aliphatic hydrocarbons such as hexadecane, heptadecane or octadecane, can result in the excess formation of diacetylated lactone-based SLs, while fatty acid esters in the growth medium lead to formation of free-acid open-chain SLs (Ashby et al., 2006; Jezierska et al., 2018). The selectivity of *S. bombicola* for incorporating C18 fatty acids into sophorolipids can be exploited to produce new sophorolipids such as a petroselinic acid diacetyl sophorolipid lactone (Delbeke et al., 2016a), where the organism is grown on petroselinic acid, a positional isomer of oleic acid where the *cis* double bond is at C6 rather than the C9 in oleic acid.

In addition to the predominant C18 form of sophorolipid produced by *S. bombicola*, it is possible to promote synthesis of C16 containing sophorolipid by the organism. Geys et al. (2018) point out that the biosynthetic pathway for sophorolipid biosynthesis has a high specificity to C18 fatty acid substrate, explaining the preference for this chain length in the biosurfactant. They were able to produce C16 sophorolipids through heterologous expression of the cytochrome P450 cyp1 (1st enzyme in the sophorolipid biosynthesis pathway) gene of *Ustilago maydis,* which introduces a higher specificity to C16 fatty acids. Feeding this modified organism with palmitic acid gives rise to C16 sophorolipid production.

Branched sophorolipids have been known for several decade (Tulloch et al., 1968), and revisited recently (Baccile et al., 2017b). These are sophorolipds with a C22 behenthic acid chain with the glycosidic bond at C13 creating two branches to the lipid chain.

Genetic modification of *S. bombicola* also offers the possibility of producing novel bolaform sophorolipid derivatives, first identified at low concentration in the wild type yeast by Price et al. (2012). Bogaert et al. (2016) have described the production of bolaform sophorolipids by an engineered *S. bombicola* with the lactone esterase gene knocked-out. Bolaform surfactants have a hydrophobic moiety with hydrophilic polar groups at each end. For sophorolipids these can be composed of either a fatty acid chain with sophorose sugar moiety on each end (a symmetrical bolaform sophoroside) or one sophorose and one carboxylic acid group at the ends (a non-symmetrical bolaform sophorolipid) (Van Renterghem et al., 2018). Delbeke et al. (2018) have extended the range of bolaform sophorolipids and sophorosides through chemical modification of diacteylated lactonic sophorolipid. This allowed them to synthesise symmetrical and non-symmetrical sophorolipids and sophorosides with longer hydrophobic linker segments and fully acetylated hydroxyl groups (peracetylated sophorolipds). Other green chemistry synthetic routes have also proven fertile ground for synthetic modification of microbially produced sophorolipids. Simple hydrogenation of the *cis* double bond of oleic sophorolipids can form the unsaturated elaidic acid derivative (Cuvier et al., 2014) leading to associated changes in self association properties. A wide range of various amino derivatives (quaternary ammonium and amine oxide derivatives) have been synthesised starting from both oleic and petroselinic sophorolipids and their biological and self-assembly properties determined (Delbeke et al., 2015, Delbeke et al., 2019; Baccile et al., 2019).

#### 3.2.2 Phase Behaviour

In common with other biosurfactants, the sophorolipids display a rich phase behaviour in solution and this aspect of their functionality has been widely studied (Zhou et al., 2004; Penfold et al., 2011; Baccile et al., 2012, Baccile et al., 2016; Dhasaiyan et al., 2013; Cuvier et al., 2014; Manet et al., 2015). At low concentrations, sophorolipids form micelles and vesicles depending on the concentration and congener type (Baccile et al., 2012, Baccile et al., 2016; Manet et al., 2015). Penfold et al. (2011) employed small angle neutron scattering (SANS) to study phase behaviour of lactonic and acidic sophorolipid at concentrations above the CMC but below 30 mM due to the low solubility of the lactonic form. Acidic sophorolipid forms small micelles in this concentration range coexisting with a low concentration of lamellar aggregates or vesicles, whilst lactonic sophorolipids form small unilamellar vesicles at concentrations up to 3 mM, large vesicles up to 7 mM and above that tubules in the range 1–30 mM. Manet et al. (2015) have looked at the structure of acidic sophorolipid in detail using scattering (SAXS and SANS) and molecular dynamics simulations. They found that sophorolipids form prolate ellipsoids at pH < 5, where they are uncharged, with an unusual core-shell type micelle structure, where there is an uneven shell (hydrophilic group) thickness, with a very thin “shell” at the ends of the long axis of the spheroid. Baccile et al. (2016) found a complex phase behaviour of sophorolipids as a function of the degree of carboxyl group ionization/pH and position. At basic pH, both sophorolipids with an oleic or stearic acid chain form small vesicles (Baccile et al., 2016). At acid pH, however, whist the oleic congener again forms micelles, the stearic congener forms a twisted ribbon type structure.

#### 3.2.3 Physicochemical Characterisation and Functionality

The surface and interfacial properties of SLs depend in part on their form, lactonic or acidic, and degree of acetylation. Sophorolipids have a relatively low CMC, similar to rhamnolipids, but their ability to lower the surface tension is less than for rhamnolipids, with a limiting surface tension around 35–40 mN/m (Van Bogaert et al., 2007) compared to 25 mN/m for rhamnolipids (Nitschke et al., 2005). Lactonic sophorolipids have a higher surface activity than the acidic form, and di-acetyl more than the mono-acetyl form, whilst the acid form is a better foamer (Van Bogaert et al., 2007). Sophorolipids have low foaming ability compared to rhamnolipids (Hirata et al., 2009) and this combined with their high surface activity makes them useful as detergents in cleaning applications for washing machines and dishwashers where foaming is disadvantageous (Hall et al., 1995; Furuta et al., 2004). Zhang et al. (2004) made various alkyl esters of sophorolipids by esterification of the fatty acid chain of sophorolipids with sodium alkoxides of varying chain length (methyl, ethyl, propyl and butyl) to modify the hydrophile-lipophile balance (HLB) in the molecules. An inverse relationship between the CMC and limiting surface tension was found, with the CMC reducing by half for each carbon added. This offers the opportunity to tune the surface properties of sophorolipids through chemical modification.

The potential of sophorolipids as additives in foods and cosmetics and the petroleum industry is evidenced by their emulsifying activity with hydrocarbon and triglyceride oils. Mixed lactonic and acidic and, especially, esterified sophorolipids are better emulsifiers of pure hydrocarbon oils and Arabian light crude than the non-ionic surfactant Triton-X (Koh et al., 2017). A similar set of studies highlighted the ability of sophorolipids alkyl esters to stabilise emulsions of lemon, almond and paraffin oil (Koh et al., 2016; Koh and Gross 2016). In these studies, the sophorolipid esters had a comparable interfacial tension lowering ability to a mixed lactonic and acetylated sophorolipid system at high concentration (1 mg/ml) for all oils, but noticeably they were able to maintain this surface activity to much lower concentrations. The various sophorolipid forms were able to emulsify each of the oils to varying degrees. The ethyl ester was the best emulsifier for paraffin oil, followed by the hexyl and then decyl (i.e., decreasing emulsifying ability with increasing alkyl chain length) whilst for the almond and lemon oil emulsions this order was reversed (decyl was the best emulsifier). However, even though the ethyl ester was the poorer emulsifier for lemon oil, the emulsions showed no separation after 1-week storage, unlike those made from the hexyl and decyl esters. Normally, smaller emulsion droplets would be associated with more stable emulsions, but in this case, it was found that the larger ethyl ester emulsions were more stable, which the authors attributed to extensive depletion flocculation in the longer chain ester emulsions due to formation of micelles from non-adsorbed surfactant in the continuous phase of the emulsion. For paraffin oil and almond oil emulsions made with the sophorolipid esters, precipitation was observed at higher surfactant concentrations due to the relatively low solubility of these molecules. A notable finding from these studies was that mixtures of lactonic and acetylated sophorolipid were poor emulsifiers in all oil systems compared to the esterified sophorolipids.

The chemical structure of SLs does not only influence the functional properties of the surfactant but also affects its biological activity (Table 1). Ma et al. (2012) studied the surface chemistry and bioactivity of lactonic and acidic SLs with different numbers of acetyl substitution, and chain length and double bond numbers of the acyl chain. They concluded that acidic SLs generally exhibited lower CMC than lactonic SLs. Moreover, lactonic SLs showed higher cytotoxic activity against Chang liver cells (Ma et al., 2012). Similar studies conducted by Shao et al. (2012) suggest that acidic SLs showed hardly any cytotoxic activity, while lactonic SLs demonstrated significant inhibitory effect on two esophageal cancer cell lines and with the effect depending on the unsaturation degree of the hydroxyl fatty acid of SLs, where the strongest cytotoxic effect was observed for one double-bond in the fatty acid moiety.

Like rhamnolipids, sophorolipids have also been investigated for use in the green synthesis of therapeutic nanoparticles (Singh et al., 2009; Shikha et al., 2020). However, unlike rhamnolipids they are able to act both as a stabilizing and reducing agent, with the reducing ability only displayed at alkaline pH (Singh et al., 2009) suggesting a critical role for the carboxyl group in acidic sophorolipids. Thus, sophorolipids offer a cleaner option for the synthesis, negating the need for toxic reducing and capping agents.

### 3.3 Mannosylerythritol Lipids

**FIGURE 2:**
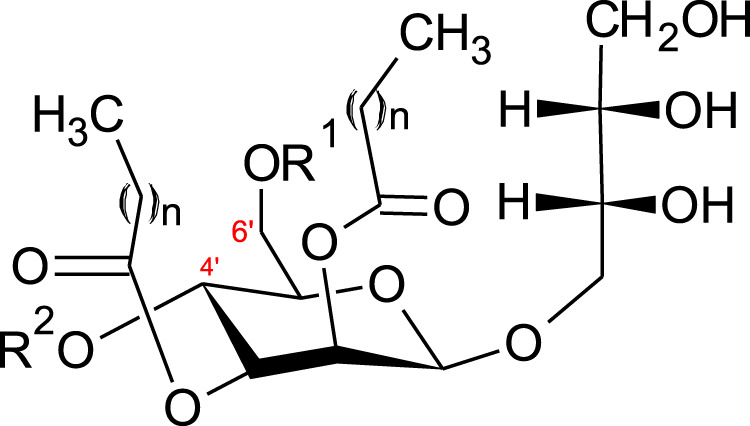
Structure of mannosylerythritol lipids (*n* = 6–10) (Konishi et al., 2007a; Yu et al., 2015). MEL-A–R^1^ = CH_3_CO, *R* ^2^ = CH_3_CO. MEL-B–R^1^ = CH_3_CO, *R* ^2^ = H. MEL-C–R^1^ = H, *R* ^2^ = CH_3_CO MEL-D–R^1^ = H, *R* ^2^ = H.

A further class of glycolipids are mannosylerythritol lipids (MEL A, -B, -C and -D), composed of a polar 4-O-β-d-mannopyranosyl-meso-erythritol attached to a non-polar fatty acid chain (Figure 2).

The hydrophilic group can be either acetylated with a short (C_2_-C_8_) or medium (C_10_-C_18_) chain fatty acid (Jezierska et al., 2018). Four main classes of MELs are defined according to the acetylation degree at C_4’_ and C_6’_ (Figure 2)–MEL-A (diacetylated), MEL-B and MEL-C (monoacetylated) or MEL-D (non-acetylated) (Yu et al., 2015; Jezierska et al., 2018).

Yeasts, mainly of the genus *Pseudozyma* (previously *Candida*) produce MELs and their structure has been proven to be strain-dependent (Jezierska et al., 2018). Fukuoka et al. (2007) concluded that the same or very similar MELs are produced by strains closely related in the phylogenetic tree. These observations were supported by Konishi et al. (2007b), who screened 13 strains for the production of MELs. A taxonomic study together with the analyses of the MELs post-fermentation allowed the authors to classify the strains into three main groups. The first group consisted of 11 strains producing all three of MEL-A, -B and -C with the most abundant fraction being MEL-A. These strains were taxonomically closely related to each other and to *Pseudozyma antarctica* and *Pseudozyma aphidis* strains, which had already been previously identified as MEL-A dominant producers (Kitamoto et al., 2001; Rau et al., 2005). The second group with only one strain produced mainly MEL-B and was related to *Pseudozyma tsukubaensis*, known as a MEL-B exclusively producing strain (Fukuoka et al., 2008; de Andrade et al., 2017). The last group, also containing only one strain related to *Pseudozyma hubeiensis* and produced predominantly MEL-C (Konishi et al., 2011).

The main substrates for MEL-producing strains are vegetable oils, giving yields of 100 g/L and higher. However, the ability of *Pseudozyma* strains to metabolize alkanes to produce MELs was also noted (Kitamoto et al., 2001; Morita et al., 2008). A very common carbon source used for fermentations is soybean oil, but often the culture medium is also enriched with small amounts of yeast extract. Factors affecting the production of MELs can be divided into four main categories 1) the effect of yeast extract, 2) nitrogen source and its concentration, 3) carbon source and its concentrations, and 4) the effect of hydrophilic precursors (such as mannose or erythritol) (Kitamoto et al., 1990; Rau et al., 2005; Konishi et al., 2008).

The physicochemical and functional characteristics of MELs depends on their structures, for example, MEL-C, which is monoacetylated would be expected to present different functionality when compared to diacetylated MEL-A. Interestingly, when the emulsification activity of MEL-C was tested, the results were as high as those of MEL-A and higher than commercial surfactants Tween80 and SDS (Konishi et al., 2008). The CMC of MEL-C was found to be 6.0 × 10^–6^ M when reducing the surface tension (ST) to 25.1mN/m, which was higher than CMC of MEL-A at 2.7 × 10^–6^ M (ST = 28.4 mN/m) and MEL-B at 4.5 × 10^–6^ M (ST = 28.2 mN/m) (Kitamoto et al., 1993; Konishi et al., 2008).

The phase behaviour of glycolipid surfactants is very complex and controlled by the acyl chain type and number, and sugar head group type and stereochemistry (Kitamoto et al., 2009). At low concentrations, MELs form micelles and vesicles of varied shape (spherical, disc and rod) (Söderman and Johansson 1999), and liquid crystalline lamellar phases at higher concentrations (Imura et al., 2007). At concentrations just above the CMC, both MEL-A and MEL-B self-associate into large unilamellar vesicles (LUV) (Imura et al., 2006). At a higher concentration, associated with a second CMC, MEL-A forms a range of liquid crystalline phases depending on temperature and concentration including sponge (L_3_), bicontinuous cubic (V_2_), and lamella (L_R_) phases (Imura et al., 2007). MEL-B, on the other hand, only exhibits one CMC above which LUVs form, with a gradual transition to multilamellar vesicles as the surfactant concentration increases (Imura et al., 2006). Konishi et al. (2008) reported that the MEL-C isolated from *Pseudozyma* KM-59 was able to self-assemble into the lyotropic liquid crystalline phases–myelines and lamella (L*α*).

In general, the formation of the lyotropic liquid crystalline phases is a favourable phenomenon of surfactants due to their potential applications as nanoparticles in drug delivery systems (Li et al., 2005; Imura et al., 2007).

### 3.4 Trehalolipids

**FIGURE 3:**
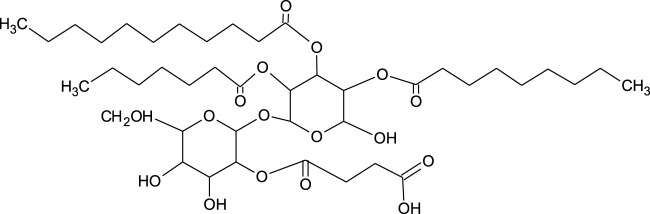
Chemical structure of trehalolipid from *Rhodococcus* sp. (Ortiz et al., 2008).

The structure of trehalolipids (TLs) is also based on a hydrophilic sugar moiety and hydrophobic fatty acid-based tail. However, there is large diversity in the fatty acid tail, being either aliphatic fatty acids or hydroxylated fatty acids with branched-chain types (*α*-branched-*β*-hydroxy fatty acids) and of assorted chain lengths (Shao 2011). The chemical structure of TLs is strongly dependent on the producing microorganisms with differences even within the same species (Figure 3) (Mnif and Dhouha 2015a). The most studied trehalolipids producers are *Rhodococcus* species along with other Gram-positive species such as *Mycobacterium*, *Nocardia, Micrococcus, Gordonia, Arthrobacter* and *Corynebacterium* (Van Hamme and Ward 2001; Ortiz et al., 2008; Shao 2011).

The amount and type of trehalose lipids produced by microorganisms also depends on the carbon source used for the microbial growth. Hvidsten et al. (2017) reported a Gram-positive bacterium belonging to the genus *Dietizia*–*Dietzia* sp. A14101 was able to utilise various hydrocarbons that directly correlated to the variety and amounts of produced trehalolipids. This clearly suggests that physicochemical characterisation of trehalolipids cannot be generalised as it depends on many factors. Marqués et al. (2009) reported functional and physicochemical characteristics of trehalolipids from *Rhodococcus erythropolis* 51T7 isolated from crude oil-contaminated soil and grown on tetradecane (2% v/v). After 72 h of cultivation, trehalolipids were extracted as a mixture of at least six components–one major and 5 minor congeners. A multi-component trehalolipid mix was also observed by Singer and Finnety (Singer and Finnerty 1990) when they grew *Rhodococcus* strain on hexadecane, resulting in a mixture of 11 components with one major and 10 minor congeners.

The CMC and surface tension reducing ability of trehalolipids are controlled by many factors. Tuleva et al. (2009) characterised the trehalose lipids extracted from the bacterium *Micrococcus luteus* BN56 grown on hexadecane, and found a CMC of 25 mg/L. The authors also detected the formation of strong emulsions even in the early stages of the cultivation of the microorganism. On the other hand, White et al. (2013) observed a CMC of 250 mg/L for trehalose lipids extracted from *Rhodococcus* sp., strain PML026. These trehalose lipids were able to make emulsions of high stability over a wide range of pH (from pH 2 to pH 10) with the highest emulsification index (EI_24_) at pH 8. The emulsifying ability (EI_24_) was independent of temperature over the range from 20 to 100°C. Similarly, Marqués et al. (2009) found a CMC of trehalolipids from *Rhodococcus erythropolis* 51T7 of 37 mg/L. The authors also presented an interesting characterisation of emulsions formed in oil-trehalolipid-water (O–TL–W) systems, where isopropyl myristate was used as the oil phase. The emulsion, which was kept at room temperature for 3 months proved to be thick, stable and the microscopic evaluation showed the emulsion particles reaching the size of 5 µm, which according to Rosen and Kunjappu (Rosen and Kunjappu 2012), classifies it as a macroemulsion. Trehalose lipid-water phase behaviour was studied as a function of temperature by polarizing microscopy. Lamellar liquid-crystalline phases were detected at low concentrations and temperature, and hexagonal phases when higher concentrations and temperature (70°C and higher) were applied. These findings indicate that there is a phase inversion from anisotropic phase of the glycolipid due to the presence of the liquid crystals (Marqués et al., 2009). These observations seem to explain the stability of the emulsions described by the authors.

In all studies discussed above, the trehalolipids showed a high propensity for surface and interfacial tension reduction. Theses promising findings suggest that trehalolipids have many characteristics that are desirable in industrial applications, but the dependence of trehalose producing organisms on long chain aliphatic carbon sources, low production yields and high costs of downstream separations are limiting factors to the scale-up of their production.

Most strains producing trehalose lipids were isolated from oil-polluted sites and were very good at degrading polluting hydrocarbon. The glycolipids produced by these oil degrading bacteria improved the bioavailability of the hydrocarbon molecules by their micellization and therefore positively affected the biodegradation. For this reason, microbially enhanced oil recovery, oil-spill bioremediation and cleaning of oil storage tanks have been the main applications of trehalolipids to date (Franzetti et al., 2010). However, their reported interaction with cell membranes, inhibition of human leukaemia cell lines, anti-fungal and anti-viral properties, and the ability to inhibit protein kinase C (PKC) activity, common in anti-tumour therapeutics, highlight their potential in medicine (Janek et al., 2018; Hirano et al., 2021).

### 3.5 Cellobiose Lipids

Cellobiose lipids have been known since the 1950’s (Haskins 1950; Lemieux et al., 1951) and were originally named ustilagic acid after the organism (the corn smut fungus *Ustilago maydis*) that was first observed to produce these. Since then, cellobiose lipids production has been identified in other fungal species (Kulakovskaya et al., 2006, Kulakovskaya et al., 2007; Golubev et al., 2008; Teichmann et al., 2011). This class of biosurfactants comprise a cellobiose (two glucose linked β1-4) hydrophilic headgroup with differing acetylation patterns, usually attached to a hydroxypalmitic acid hydrophobic moiety (Eveleigh et al., 1964). Various other congeners have been identified including di and tri-hydroxyhexadecanoic acid chains, dehydroyxy congeners, additional acetyl groups and octanoic acid replacing the palmitic acid in the hydroxylated lipid chains (Spoeckner et al., 1999; Teichmann et al., 2007, Kulakovskaya et al., 2011). Bolaform cellobiose lipids have also been observed as fermentation products with some organisms (Puchkov et al., 2002; Morita et al., 2011).

### 3.6 Polyol Lipids

Polyol lipids (PLs) belong to the class of extracellular fungal glycolipid biosurfactants (EFGB), however, structurally they are different from sophorolipids and mannosylerythritol lipids by not having a sugar component as the hydrophilic moiety but instead have a polyol as a polar group. Only two types of PLs have been found to date, polyol esters of fatty acids (PEFA) and liamocins (Garay et al., 2018).

#### 3.6.1 Polyol Esters of Fatty Acids

Polyol esters of fatty acids (PEFA) are produced mainly by the yeasts of the genus *Rhodotorula* (Garay et al., 2018). As for the other microorganism-produced surfactants, the structure and yields of the produced PEFAs can significantly vary depending on the secreting strain.

**FIGURE 4:**
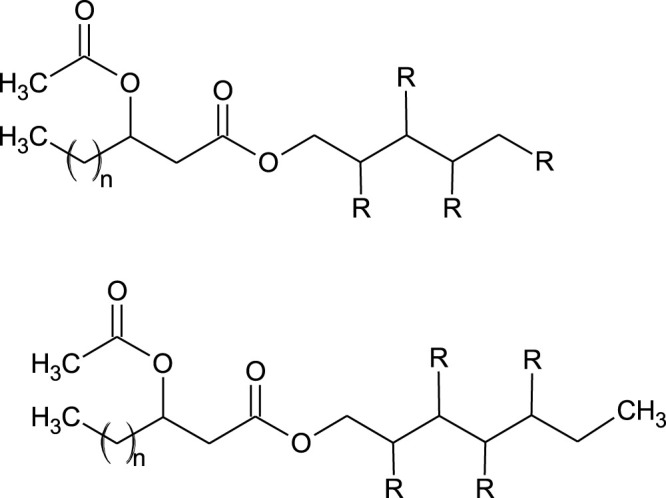
General structure of PEFA (Garay et al., 2018). Where: R = OH or acetyl *n* = 8, 10, 12, 14, 16.

Nineteen PEFAs have been identified to date, allowing a general summary of the structures to be defined (Cajka et al., 2016; Garay et al., 2017; Garay et al., 2018). Briefly, the molecules consist of a polyol head group (usually D-mannitol or D-arabitol but other polyols are also possible) esterified to an acetylated (R)-3-hydroxyacyl fatty acid via the carboxyl end (Cajka et al., 2016; Garay et al., 2017; Garay et al., 2018). Figure 4 presents the general structure of the PEFAs with several possible variations.

The optimal conditions for PEFA production are: temperature between 24 and 27°C, pH = 6.3–6.5, and a medium known as Medium A (Garay et al., 2017; Garay et al., 2018). The carbon source most commonly used is glucose, however, glycerol, sucrose, molasses and plant-based hydrolysates can also be used (Garay et al., 2017; Garay et al., 2018).

Based on structural and physicochemical studies, PEFA seem to have potential as biosurfactants. Lyman et al. (2018) report that *Rhodotorula taiwanensis* MD1149 produces the hypoacetylated PEFA (0–4 acetylation modifications) with increased surface-active properties–mainly ST reduction to approximately 32 mN/m (Lyman et al., 2018). The isolates of *R. aff. paludigena* UCDFST 81–84 and *R. babjevae* UCDFST 04–877 were observed to reduce the ST down to 33.3 and 30.4 mN/m respectively and additionally, *R. aff. paludigena* UCDFST 81–84 isolate showed antifoam activity, which is a promising feature for the industrial application of this substance (Garay et al., 2018).

#### 3.6.2 Liamocins

Liamocins are polyol lipids produced by various strains of Aureobasidium pullulans. The term “liamocins” was introduced by Price et al. (2013), despite the molecules being known from 1964 (Garay et al., 2018).

Structural studies have shown liamocins have a single, partially O-actetylated polyol polar group, and three or four 3,5-dihydroxy-decanoic ester group polyester tails (three in liamocin A1 and A2 and four in liamocin B1 and B2) (Price et al., 2013, Price et al., 2017).

The selection of strain and culture conditions can lead to the formation of many diverse structures of liamocins, as observed by Leathers et al. (2015). Price et al. (2017) reported a variety of different head groups of liamocins (D-galactitol, D-sorbitol, D- and L-arabitol, D-xylitol, L-threitol and glycerol), when varied polyols were used in the growth media. Interestingly, no such dependence was shown when different sugars were used in the culture medium.

In recent years liamocins were reported to exhibit antibacterial activity as well as being inhibitors of some cancer cell lines (Manitchotpisit et al., 2014; Leathers et al., 2016).

Little is still known about the physicochemical properties of liamocins and their possible application in the industry. Liamocins isolated from Aureobasidium pullulans CU 43 were reported to be fluorescent and able to reduce the ST to 27 mN/m (Manitchotpisit et al., 2011). Kim et al. (2015) reported a glycerol-based liamocin (glycerol-liamocin) from Aureobasidium pullulans L3-GPY, which decreased the ST to 31.5 mN/m at the concentration of 1.5 mg/L.

The abovementioned findings suggest that liamocins have the potential of being used as biosurfactants, but a further, detailed investigation of their functionality is needed.

## 4 Conclusion

Glycolipids are a class of biosurfactants of high potential, not only in bioremediation and clean-up strategies, or as household-care agents, but also as very good materials for food and medical applications. Most importantly, they are all of natural origin and can mostly be produced using particular organic-wastes (waste glycerol as an example). What is more, the optimised conditions for the production of glycolipids are easy to obtain and the costs of the production may be lower when compared to the chemically-derived surfactants.
